# Travis County Medical Association—Delegates

**Published:** 1886-03

**Authors:** 


### TRAVIS COUNTY MEDICAL ASSOCIATION.

AT the regular meeting of this Association, held in Austin March 4th inst., the following delegates were elected: To the American Medical Association at St. Louis, Drs. R. M. Swearingen, T. D. Wooten and F. E. Daniel; to State Medical Association at Dallas, Drs. J. W. McLaughlin, R. M. Swearingen, T. J. Bennett, B. E. Hadra and F. E. Daniel.

